# Long‐term safety of ultrathin bioabsorbable‐polymer sirolimus‐eluting stents versus thin durable‐polymer drug‐eluting stents in acute coronary syndrome: A systematic review and meta‐analysis

**DOI:** 10.1002/clc.24139

**Published:** 2023-09-03

**Authors:** Fadong Li, Shen Wang, Yue Wang, Can Wei, Yue Wang, Xinyan Liu, Shuaifeng Sun, Wenxin Zhao, Pengrong Guo, Xiaofan Wu

**Affiliations:** ^1^ Department of Cardiology, Beijing Anzhen Hospital Capital Medical University Beijing China; ^2^ Department of Pathophysiology Harbin Medical University Harbin China

**Keywords:** acute coronary syndrome, bioabsorbable‐polymer, drug‐eluting stents, ultrathin‐strut

## Abstract

**Background:**

Because of the advancement of bioabsorbable polymers and thinner struts, bioabsorbable‐polymer sirolimus‐eluting stents (BP‐SES) with ultrathin struts may be related to superior performance when compared to durable‐polymer drug‐eluting stents (DP‐DES) with thin struts. Nonetheless, the long‐term safety of ultrathin BP‐SES in acute coronary syndrome (ACS) remains unknown.

**Methods:**

We sought to assess the long‐term safety of ultrathin BP‐SES in ACS patients, conducting a thorough meta‐analysis of all relevant trials drawing a comparison between ultrathin BP‐SES and contemporary thin DP‐DES. Target lesion failure (TLF), which includes cardiac death (CD), target‐vessel myocardial infarction (TV‐MI), and clinically driven target lesion revascularization (CD‐TLR) was considered the primary endpoint. Multiple databases comprising Embase, MEDLINE, Cochrane Library, and Pubmed were all thoroughly searched.

**Results:**

There were seven randomized controlled trials included in our study with 7522 randomized patients with ACS (BP‐SES = 3888, DP‐DES = 3634). TLF occurred in 371 (9.5% in BP‐SES) and 393 (10.8% in DP‐DES) patients, respectively, across a 40.7‐month weighted mean follow‐up, with no statistically significant group differences (risk ratio [RR]: 0.87; 95% confidence interval [CI]: 0.73–1.04; *p* = .12). Furthermore, no significant differences in cardiac death (RR: 0.96; 95% CI: 0.68–1.35; *p* = .81), TV‐MI (RR: 0.63; 95% CI: 0.36–1.10; *p* = .10) and CD‐TLR (RR: 0.77; 95% CI: 0.46–1.29; *p* = .32) were detected between two groups.

**Conclusion:**

During a follow‐up of 40.7 months, ultrathin BP‐SES and thin DP‐DES had a comparable risk of TLF and its individual components (CD, TV‐MI, and CD‐TLR), indicating that ultrathin BP‐SES held at least the same safety and efficiency as thin DP‐DES presented in patients with ACS.

## Graphical abstract

A meta‐analysis including seven large randomized controlled trial indicated that ultrathin BP‐SES and thin DP‐DES had a similar risk of TLF and its individual components (CD, TV‐MI, and CD‐TLR) in ACS patients during a long‐term follow up.

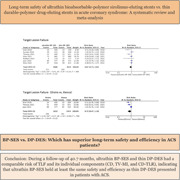

## 1 INTRODUCTION

Presently, patients presenting with acute coronary syndrome comprising high‐risk unstable angina (UA), ST‐segment elevation myocardial infarction (STEMI), or non‐ST‐segment elevation myocardial infarction (NSTEMI), are advised to be treated by percutaneous coronary intervention (PCI).^1^, ^2^ After years of development on stents, drug‐eluting stents (DES) are found to be linked with a relatively low risk of recurring target‐vessel revascularization by contrast with prior bare‐metal stents (BMS).^3^ Additionally, due to the advancement of biodegradable polymers and thinner metallic stent platforms, new‐generation DES may have conserved or even enhanced safety and efficacy compared to prior‐generation DES. Patients with acute coronary syndrome (ACS) have a significantly elevated probability of recurring stent‐related adverse events.^4^ by contrast with patients presenting with chronic coronary syndrome (CCS) because of delayed arterial healing as well as aggravated inflammation response.^5^, ^6^ The latest‐generation DES comprising ultrathin‐strut bioabsorbable‐polymer sirolimus‐eluting stents (BP‐SES), are considered to be beneficial to endothelialization and contribute to mitigating chronic inflammation, arterial injury, and thrombogenicity.^7^ It may be of great benefit to apply ultrathin BP‐SES for ACS patients due to the valued improvements and modifications. A prior study observed that the newer ultrathin DES contributed to improving long‐term outcomes by contrast with thicker‐strut DES among patients with coronary artery disease at a 2.5‐year follow‐up.^8^ Additionally, several studies evaluated the performance of ultrathin BP‐SES and thin durable‐polymer drug‐eluting stents (DP‐DES), shedding light on their clinical safety.^9^, ^10^ However, the existing studies primarily focused on short‐term or midterm clinical outcomes and paid limited attention to patients with ACS. In view of this knowledge gap and inspired by recent reports of several large clinical trials,^11^, ^12^ we conducted a meta‐analysis of seven randomized controlled trials (RCTs) to provide more evidence for the long‐term safety of ultrathin BP‐SES versus contemporary thin DP‐DES in patients with ACS.

## 2 MATERIALS AND METHODS

The current study including seven RCTs was conducted in adherence to the Preferred Reporting Items for Systematic reviews and Meta‐Analyses (PRISMA) guidelines^13^ and was registered in advance at PROSPERO (registration ID: CRD42023411378).

### 2.1 Literature search

Multiple databases comprising Embase, MEDLINE, Cochrane Library, and Pubmed were all thoroughly searched for all RCTs from inception to February 15, 2023, comparing long‐term outcomes of ultrathin BP‐SES to those of contemporary DP‐DES with thin struts among patients presenting with ACS. To guarantee that all relevant studies were included during the literature search, the following combinations of relevant medical terms were used: “bioabsorbable polymer,” “biodegradable polymer,” “bioresorbable polymer,” “durable polymer,” “ultrathin strut,” “sirolimus‐eluting,” “drug‐eluting stents,” “BP‐SES,” “DP‐DES,” “DES,” and “acute coronary syndrome,” “myocardial infarction,” “unstable angina,” “ACS,” “STEMI,” “NSTEMI” and “UA.” Full reference lists of enrolled studies were manually scanned by our researchers to acquire more eligible trials. During the literature search, no constraints on language or sample size were set. Two independent researchers (F. D. L. and S. W.) conducted the search process, screening the titles, abstracts as well as full text when necessary and selecting eligible studies according to predefined inclusion criteria. After consulting with another researcher (Y. W.), disagreements were settled.

### 2.2 Inclusion and exclusion criteria

We only included RCTs that made comparisons between ultrathin BP‐SES and thin DP‐DES in patients who presented with ACS and had follow‐up reports for at least 1 year. Ultrathin struts were defined as those with thickness less than 70 μm while thin struts were defined as those with thickness between 70 and 100 μm. Only the reports with the longest follow‐up were included when there were several reports of the same clinical trial that had different follow‐up durations. Due to the relatively high possibility of biased results, observational and retrospective studies were discarded.

### 2.3 Data extractions and quality assessments

Two researchers (F. D. L. and S. W.) independently conducted the process of data extraction, and a third researcher (Y. W.) verified the final result. From each included study, predefined data were extracted: authors, study design, year of publication, follow‐up duration, number of enrolled patients, entry and exclusion criteria, stent type, baseline clinical characteristics, and clinical outcomes. The quality of all included RCTs was assessed by three researchers (F. D. L., S. W., and Y. W.) with Cochrane Collaboration Risk of Bias tool.

### 2.4 Study endpoints

Target lesion failure (TLF), a composite of cardiac death (CD), target‐vessel MI (TV‐MI), and clinically driven target lesion revascularization (CD‐TLR) was considered the primary endpoint. The secondary endpoints were the individual components of TLF. Trial‐specific definition was used when there were minor variations from the standard definition of TLF.

### 2.5 Statistical analysis

*I* ^2^ statistic was used to assess the statistical heterogeneity between RCTs. Depending on the resulting value of *I* ^2^, it was classified as low (≤25%), moderate (between 25% and 75%), or high (≥75%) heterogeneity. In cases where there was moderate or high heterogeneity, we performed random‐effects models. Conversely, when the heterogeneity was low, fixed‐effects models were used instead. The evaluation of clinical outcomes was conducted using relative risk ratios (RRs) accompanied by their corresponding 95% confidence intervals. To present these findings in a visually informative manner, forest plots were utilized. Sensitivity analyses were conducted by excluding each study in turn. To evaluate the publication bias of studies, funnel plots were visually inspected. Review Manager 5.4 software was utilized to compute all data involved. Statistical significance was considered if a *p* value for hypothesis test was less than .05.

## 3 RESULTS

### 3.1 Search strategy

**Figure 1:**
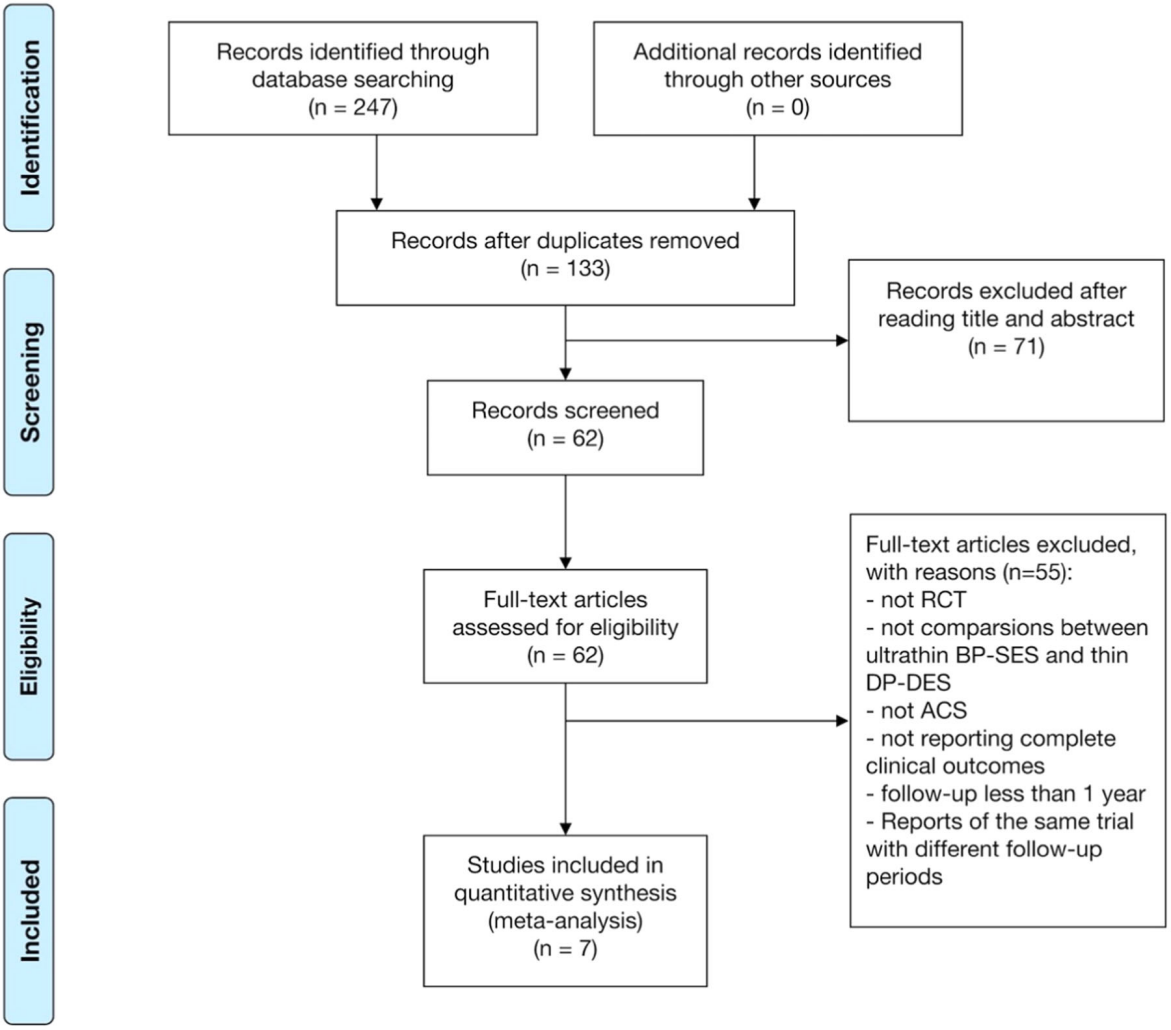
Flow diagram of the search strategy for systematic review and meta‐analysis.

After the removal of duplicate records (*n* = 114), we screened the titles and abstracts of 133 records. Furthermore, 62 studies of the above records were assessed for eligibility by reviewing the full text and seven studies were included ultimately. Studies were excluded mainly with reasons that they did not make comparisons between ultrathin BP‐SES and thin DP‐DES in patients with ACS, and full reasons for exclusion are outlined in Figure 1. Finally, 7522 patients with ACS undergoing PCI with ultrathin BP‐SES (*n* = 3888) or thin DP‐DES (*n* = 3634) from seven RCTs were eligible for enrollment in the current meta‐analysis^12^, ^14^, ^15^, ^16^, ^17^, ^18^, ^19^ (Figure 1 and Table 1).

**Table 1:** Characteristics of included studies.

Author	Design	Year	ACS patients enrolled (n)	Proportion of ACS patients (%)	Mean Age^a^ (years)	Follow‐up (months)	Stent Type	Primary outcomes
Robbert J. de Winter et al.	RCT	2022	Eg: 119 Cg: 117	58.1	Eg: 66 (IQR:58‐72) Cg: 65 (IQR: 58‐72)	36	Eg: Supraflex Cg: Xience	Device‐oriented composite endpoint of cardiac death, target vessel myocardial infarction, and clinically indicated target lesion revascularisation
Juan F. Iglesias et al.	RCT	2022	Eg: 577 Cg: 554	53.4	Eg: 64.6 ± 12.3 Cg: 64.8 ± 12.3	60	Eg: Orsiro Cg: Xience Prime/Xpedition	Target lesion failure, a composite of cardiac death, target‐vessel myocardial infarction, or clinically indicated target lesion revascularization
Eline H. Ploumen et al.	RCT	2022	Eg: 818 Cg: 815	69.7	Eg: 64.2 ± 10.7 Cg: 63.6 ± 10.9	60	Eg: Orsiro Cg: Resolute Integrity	Target vessel failure, a composite of cardiac death, target vessel‐related myocardial infarction, or clinically indicated target vessel revascularisation
Eline H. Ploumen et al.	RCT	2021	Eg: 885 Cg: 880	70.9	Eg: 63.9 ± 11.2 Cg: 64.1 ± 10.9	36	Eg: Orsiro Cg: Resolute Onyx	Target vessel failure, a composite of cardiac death, target vessel‐related myocardial infarction, or target vessel revascularization
Thomas Pilgrim et al.	RCT	2021	Eg: 649 Cg: 651	100.0	Eg: 62.2 ± 11.8 Cg: 63.2 ± 11.8	24	Eg: Orsiro Cg: Xience Xpedition/Alpine	Target lesion failure, a composite of cardiac death, target vessel myocardial reinfarction, and clinically indicated target lesion revascularization
Kuniaki Takahashi et al.	RCT	2020	Eg: 414 Cg: 408	58.8	Eg: 66.4 ± 10.7 Cg: 66.3 ± 10.7	36	Eg: MiStent Cg: Xience	Device oriented composite endpoint, defined as occurrence of either cardiac death, myocardial infarction not clearly attributable to a nontarget vessel, or clinically indicated target lesion revascularisation
Ariel Roguin et al.	RCT	2018	Eg: 426 Cg: 209	50.7	Eg: 63.1 ± 10.8 Cg: 63.2 ± 11.2	12	Eg: Orsiro Cg: Xience	Target lesion failure, a composite of cardiovascular death, target vessel‐related myocardial infarction, or ischemia‐driven target lesion revascularisation

Abbreviations: Cg, control group (thin‐strut durable polymer drug‐eluting stents); Eg, experimental group (ultrathin‐strut bioabsorbable polymer sirolimus‐eluting stents).

^a^ Mean age (±SD) or median age (IQR).

### 3.2 Characteristics of enrolled studies and risk of bias assessment

The weighted mean follow‐up duration was 40.7 months with the shortest follow‐up duration being 1 year and the longest 5 years. Table 1 presents an overview of major characteristics of enrolled studies, while Table 2 depicts the baseline characteristics with no significant difference between groups (ultrathin BP‐SES or thin DP‐DES) in individual trials. In addition, detailed entry and exclusion criteria of each included study are presented in Supporting Information: Table S1. Three kinds of ultrathin‐strut BP‐SES from different manufacturers were utilized in included RCTs, comprising Orsiro (five studies), MiStent (one study), and Supraflex (one study) while thin‐strut DP‐DES used in these studies were concluding Xience (five studies), Resolute Integrity (one study), and Resolute Onyx (one study). The principal features of the stents mentioned above are outlined in Supporting Information: Table S2. Furthermore, interaction analyses between stent type (ultrathin BP‐SES or thin DP‐DES) and clinical presentation (ACS or not) for the primary endpoint in enrolled studies are presented in Supporting Information: Table S3. Supporting Information: Table S4 provides a detailed judgment of risk of bias for the enrolled studies (also shown in Supporting Information: Figure S1).

**Table 2:** Baseline characteristics of included studies.

Study (Author)	Age (years)	Male (%)	Current smoker (%)	Hypertension (%)	Diabetes mellitus (%)	Hyperlipidemia (%)	Previous MI(%)	Previous PCI (%)	Previous CABG (%)
Robbert J. de Winter et al. BP‐SES/DP‐DES	66/65	75.8/76.5	24.5/24.1	65.3/66.1	21.8/24.9	61.8/60.2	18.9/17.9	24.3/21.4	4.6/7.7
Juan F. Iglesias et al. BP‐SES/DP‐DES	64.7/64.6	77.5/77.8	34.4/35.2	61.4/61.8	19.2/19.8	59.8/60.9	13.6/15.3	17.4/20.3	6.1/6.8
Eline H. Ploumen et al. 2022 BP‐SES/DP‐DES	64.2/63.6	73.0/72.0	30.0/31.0	47.0/47.0	18.0/18.0	40.0/38.0	18.0/21.0	18.0/17.0	7.0/8.0
Eline H. Ploumen et al. 2021 BP‐SES/DP‐DES	63.9/64.1	76.1/76.1	30.7/20.6	53.2/49.8	20.1/20.9	46.4/45.4	16.5/15.6	22.3/21.1	7.8/6.4
Thomas Pilgrim et al. BP‐SES/DP‐DES	62.2/63.2	79/73	45/39	43/46	11/13	47/47	4/4	4/5	0.3/1
Kuniaki Takahashi et al. BP‐SES/DP‐DES	66.4/66.3	70/74	27/26	72/75	9/9	61/60	27/28	34/36	7/10
Ariel Roguin et al. BP‐SES/DP‐DES	63.1/63.2	74.2/70.4	29.5/26.0	76.7/79.8	34.0/36.9	76.1/82.5	28.7/26.2	29.0/25.8	6.9/5.0

Abbreviations: CABG, coronary artery bypass grafting; MI, myocardial infarction; PCI, percutaneous coronary intervention.

### 3.3 Primary endpoint

**Figure 2:**
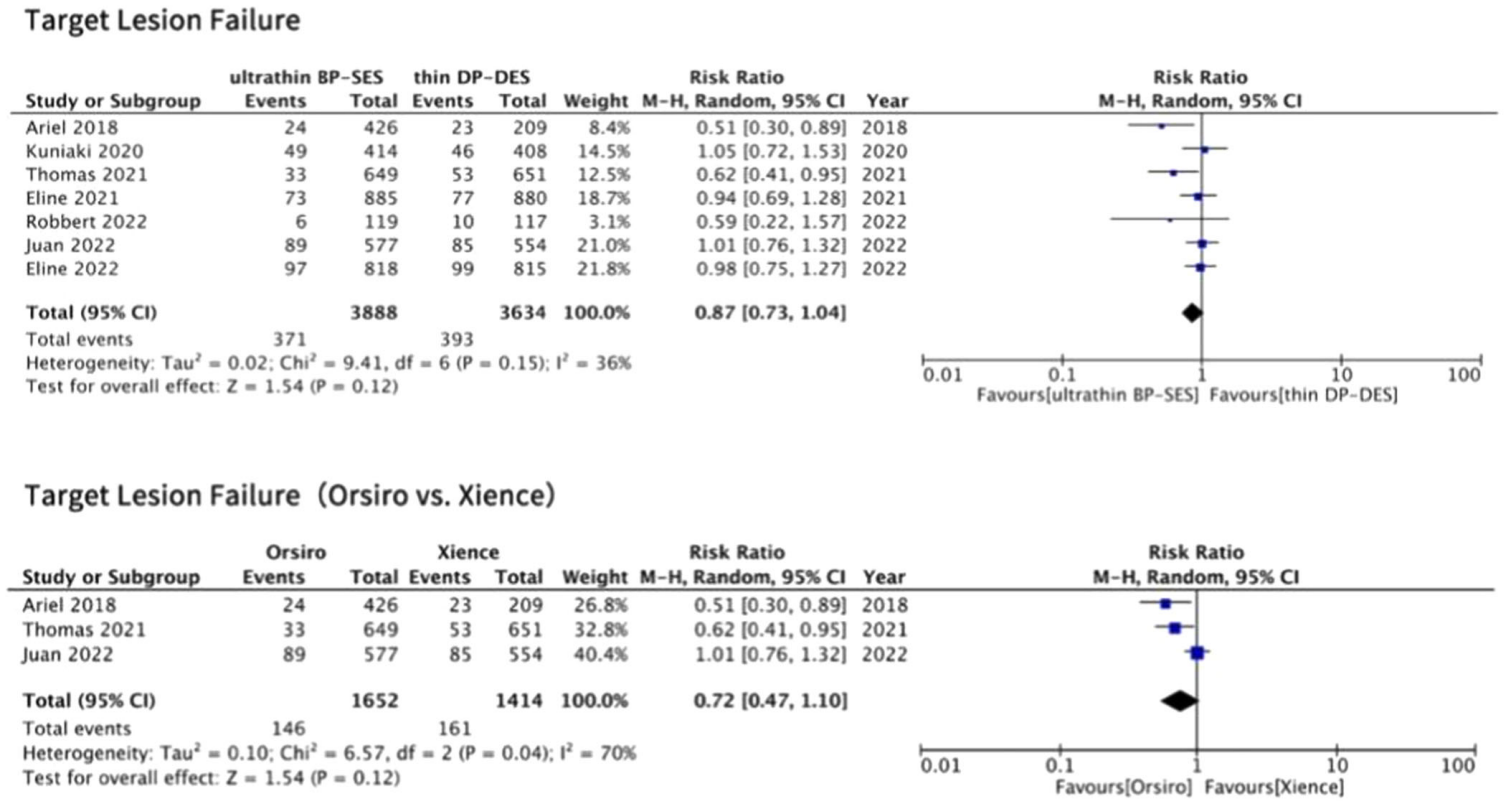
Risk of target lesion failure (TLF) at a long‐term follow‐up. BP‐DES, bioabsorbable‐polymer sirolimus‐eluting stent; CI, confidence interval; DP‐DES, durable‐polymer drug‐eluting stent.

Clinical follow‐up outcomes regarding TLF occurrence were accessible from all seven RCTs, involving a total of 7522 patients enrolled. Our analysis indicated that experimental and control groups did not differ significantly in the risk of TLF among ACS patients (risk ratio [RR]: 0.87; 95% CI: 0.73–1.04; *p* = .12) (as shown in Figure 2). The enrolled studies displayed moderate heterogeneity (*I* ^2^ = 36%) with no signs of publication bias detected (Supporting Information: Figure S2). Sensitivity analyses were conducted by excluding each trial in turn and the primary outcome of TLF remained to present no difference between the two groups (Supporting Information: Table S5). Notably, we also conducted a head‐to‐head comparison between Orsiro and Xience to minimize the influence caused by different stent types in each group (ultrathin BP‐SES or thin DP‐DES). And the outcome of TLF remained consistent, indicating no difference between groups (RR: 0.72; 95% CI: 0.47–1.10; *p* = .12) (Figure 2).

### 3.4 Secondary endpoints

**Figure 3:**
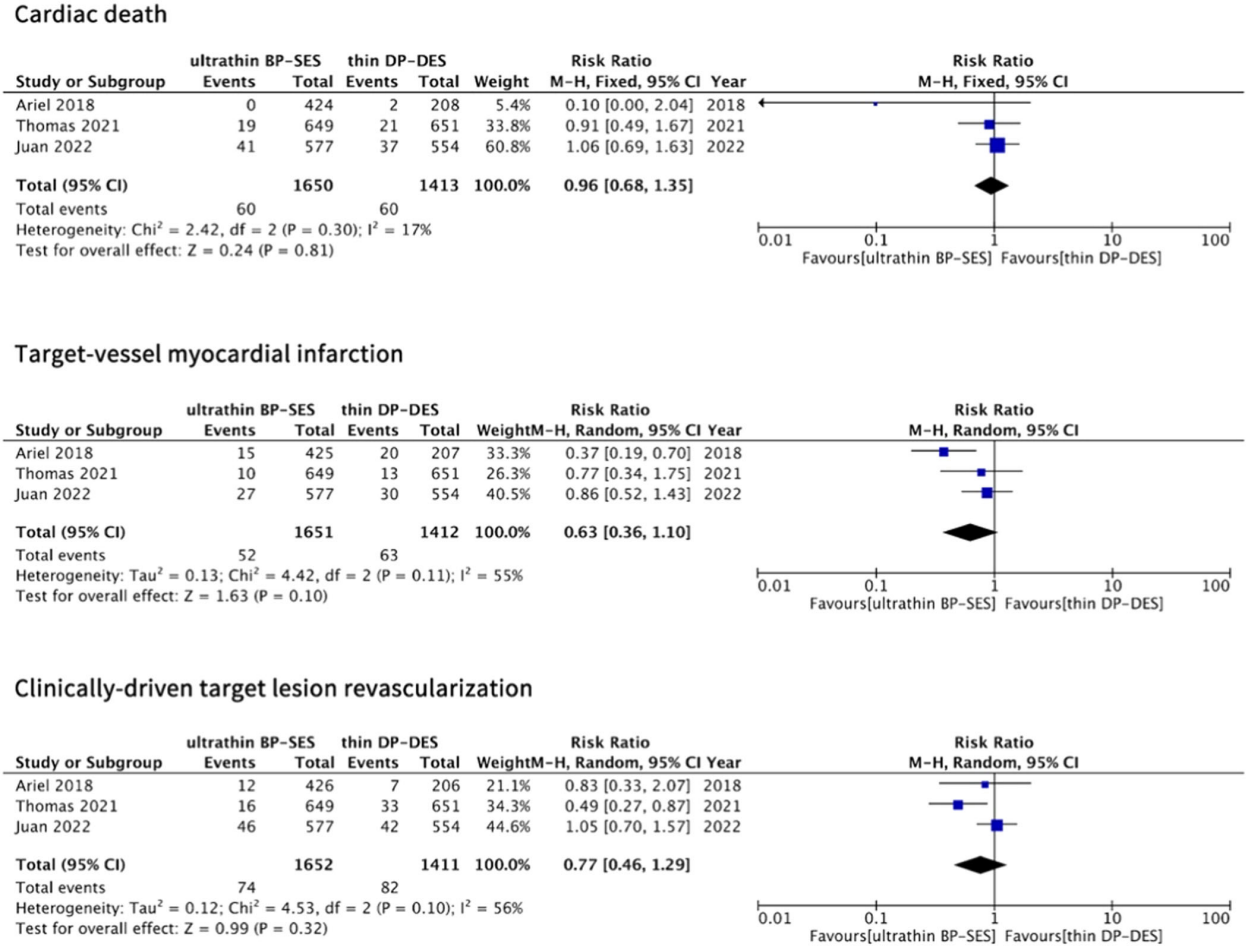
Risk of cardiac death (CD), target‐vessel myocardial infarction (TV‐MI), and clinically‐driven target lesion revascularization (CD‐TLR) at a long‐term follow‐up. BP‐DES, bioabsorbable‐polymer sirolimus‐eluting stent; CI, confidence interval; DP‐DES, durable‐polymer drug‐eluting stent.

The clinical outcomes of CD, TV‐MI, and CD‐TLR could be obtained from three studies with 3063 patients. And no significant differences between the two groups were shown in terms of the risk of CD (RR: 0.96; 95% CI: 0.68–1.35; *p* = .81), TV‐MI (RR: 0.63; 95% CI: 0.36–1.10; *p* = .10), or CD‐TLR (RR: 0.77; 95% CI: 0.46–1.29; *p* = .32) (as depicted in Figure 3). Low or moderate heterogeneities (*I* ^2^ = 17%, 55%, and 56%, respectively) were observed between the studies.

## 4 DISCUSSION

As far as we could tell, our present meta‐analysis of seven RCTs, including 7522 patients appears to be the primary study to investigate long‐term outcomes of ultrathin BP‐SES versus preceding thin DP‐DES in patients presenting with ACS. The main finding of our study is that patients with ACS had a similar risk of TLF at a long‐term follow‐up after undergoing PCI with either ultrathin BP‐SES or thin DP‐DES. Additionally, no statistically significant differences were detected in the occurrence of CD, TV‐MI, and CD‐TLR between the two groups in our analysis as well.

The second‐generation DES have been widely utilized in patients who underwent PCI during past years due to the excellent clinical performance in contrast to BMS. Nonetheless, the risk of very‐late stent‐related adverse events among patients after PCI showed no significant difference among second‐generation DES, first‐generation DES, and BMS.^20^ Recent improvements in DES such as ultrathin strut and biodegradable polymer could be an effective approach to present superior long‐term clinical outcomes. There were studies holding the viewpoint that biodegradable polymers could contribute to reducing chronic inflammatory responses^21^ and hypersensitivity reactions^22^ compared with permanent ones. And a previous study indicated that thinner stent platforms were involved in less stent thrombogenicity compared to thicker stent platforms, which may be attributed to improved endothelialization, less arterial injury, less local flow disturbance, and decreased inflammatory response.^7^ Also, a randomized clinical trial illustrated that the improvements on stent platform thickness had outstanding benefits for decreasing the risk of restenosis among patients after PCI.^23^ Despite this, further study maintains its necessity to confirm the correspondence between thinner struts and long‐term clinical outcomes in patients.

Ultrathin BP‐SES potentially has advantages with regard to decreasing stent‐related adverse events due to the specific biodegradable polymers and tinner metallic stent platforms. A study published recently reported that BP‐DES and DP‐DES exhibited comparable performance in clinical practice regardless of procedure complexity.^24^ And a preceding meta‐analysis reported that DES with ultrathin struts substantially decreased the risk of TLF by contrast with prior‐generation DES at an average follow‐up of 2.5 years.^8^ However, there are various properties of DES designs including but not limited to strut thickness, polymer degradation time, polymer composition, stent platform geometry, and stent deliverability, which might exert influences on clinical outcomes among patients.

Our study focused on the particular comparison between BP‐SES with ultrathin struts and DP‐DES with thin struts, having an important difference with prior meta‐analysis that generally compared ultrathin‐strut DES to second‐generation DES neglecting polymer and platform characteristics. Furthermore, our analysis had an average follow‐up duration of 40.7 months, which offered more statistical power to demonstrate long‐term safety of ultrathin BP‐SES for patients who need to undergo PCI. There was a meta‐analysis suggesting that ultrathin‐strut DES (including BP‐SES) was relevant to a lowered risk of TLF by contrast to prior thicker DES at a 1‐year follow‐up, which is mainly owing to a significantly decreased risk of CD‐TLR.^25^ Similarly, in the current meta‐analysis, we observed that TLF occurred in 9.5% (BP‐SES) and 10.8% (DP‐DES) of ACS patients, respectively at a 40.7‐month follow‐up. In spite of no statistical significance achieved, ultrathin‐strut BP‐SES could be confirmed to hold at least the same safety and efficiency compared with thin‐strut DP‐DES over a long period. In addition, our meta‐analysis paid attention to patients with ACS due to the higher risk for recurrent stent‐related adverse events instead of including all‐comer patients. Differing from patients with CCS, patients with ACS are involved in increased prothrombotic and inflammatory response resulting in delayed arterial healing^5^, ^6^ and thus have a raised risk for long‐term cardiovascular adverse events. Long‐term benefits of ultrathin‐strut DES in ACS have recently been further studied, indicating a trend to improve 1‐year outcomes but presenting a marginal advantage statistically.^25^ And our meta‐analysis indicated a comparable risk of TLF in patients with ACS in a long period (more than 3 years) between the two groups, which was aligned with the subgroup analysis of a previous IPD meta‐analysis of five RCTs.^10^ Remarkably, a recent study based on optical coherence tomography (OCT) found that Orsiro ultrathin BP‐SES (O‐SES) exhibited better coverage of struts and thinner neointima compared to Xience thin DP‐EES (X‐EES), which could also support the safety and efficiency of ultrathin BP‐SES by means of intravascular imaging.^26^ It remains necessity for further examination with additional clinical evidence whether BP‐SES will exhibit superior performance over thin DP‐DES during an even more extended follow‐up duration. Additionally, in our current meta‐analysis, none of the risks of CD, TV‐MI, and CD‐TLR was statistically different between the two groups as well. However, underlying differences in CD, TV‐MI as well as CD‐TLR between the two groups could not be excluded.

## 5 LIMITATIONS

We acknowledged some limitations in our study. First, due to the limited RCTs that reported long‐term outcomes of ACS patients who underwent PCI with ultrathin bioabsorbable‐polymer DES, the studies enrolled in our analysis did not show a conformity of follow‐up, which varied between 1 year and 5 years. Second, due to limited studies exclusively focusing on ACS patients, we extracted relevant data on clinical outcomes of ACS patients from the original studies. We attempted to reach out to the principal authors of these original studies to obtain comprehensive information specifically related to ACS patients' clinical outcomes but unfortunately receive no response. Third, it may exert an influence on the final conclusion of individual components of TLF that restricted corresponding follow‐up data were available. More studies in the future are needed to determine the difference between ultrathin BP‐SES and contemporary thin DP‐DES in terms of the risk of CD, TV‐MI, and CD‐TLR. Finally, our analysis was unable to obtain individual patient data from each selected trial, as with any other study‐level meta‐analysis. As a result, statistical procedures such as multivariable and subgroup analyses could not be applied in our study to illustrate the difference in baseline characteristics.

## AUTHOR CONTRIBUTIONS

Fadong Li, Xinyan Liu, Wenxin Zhao, and Pengrong Guo: Concept and design. Yue Wang: supervision and critical review. Fadong Li and Yue Wang: analysis and interpretation. Fadong Li and Shen Wang: drafting the manuscript and literature search. Can Wei and Shuaifeng Sun: revising the manuscript. All authors contributed to the funding and approved the submitted version.

## CONFLICT OF INTEREST STATEMENT

The authors declare no conflict of interest.

## Supporting information

Supporting information.

## Data Availability

All relevant data generated in this study are available in this article.
