# Supplementary material for: Long‐term safety of ultrathin bioabsorbable‐polymer sirolimus‐eluting stents versus thin durable‐polymer drug‐eluting stents in acute coronary syndrome: A systematic review and meta‐analysis
Source: Clin Cardiol. 2023 Sep 3;46(12):1465–73. doi: 10.1002/clc.24139 (PMC10716332; doi:10.1002/clc.24139)
Supplement: Supplementary file 1 — Supporting information. [file CLC-46-1465-s001.docx]

Supplementary Material

Long-term safety of ultrathin bioabsorbable-polymer sirolimus-eluting stents vs. thin durable-polymer drug-eluting stents in acute coronary syndrome: A systematic review and meta-analysis

1. Supplementary Figures


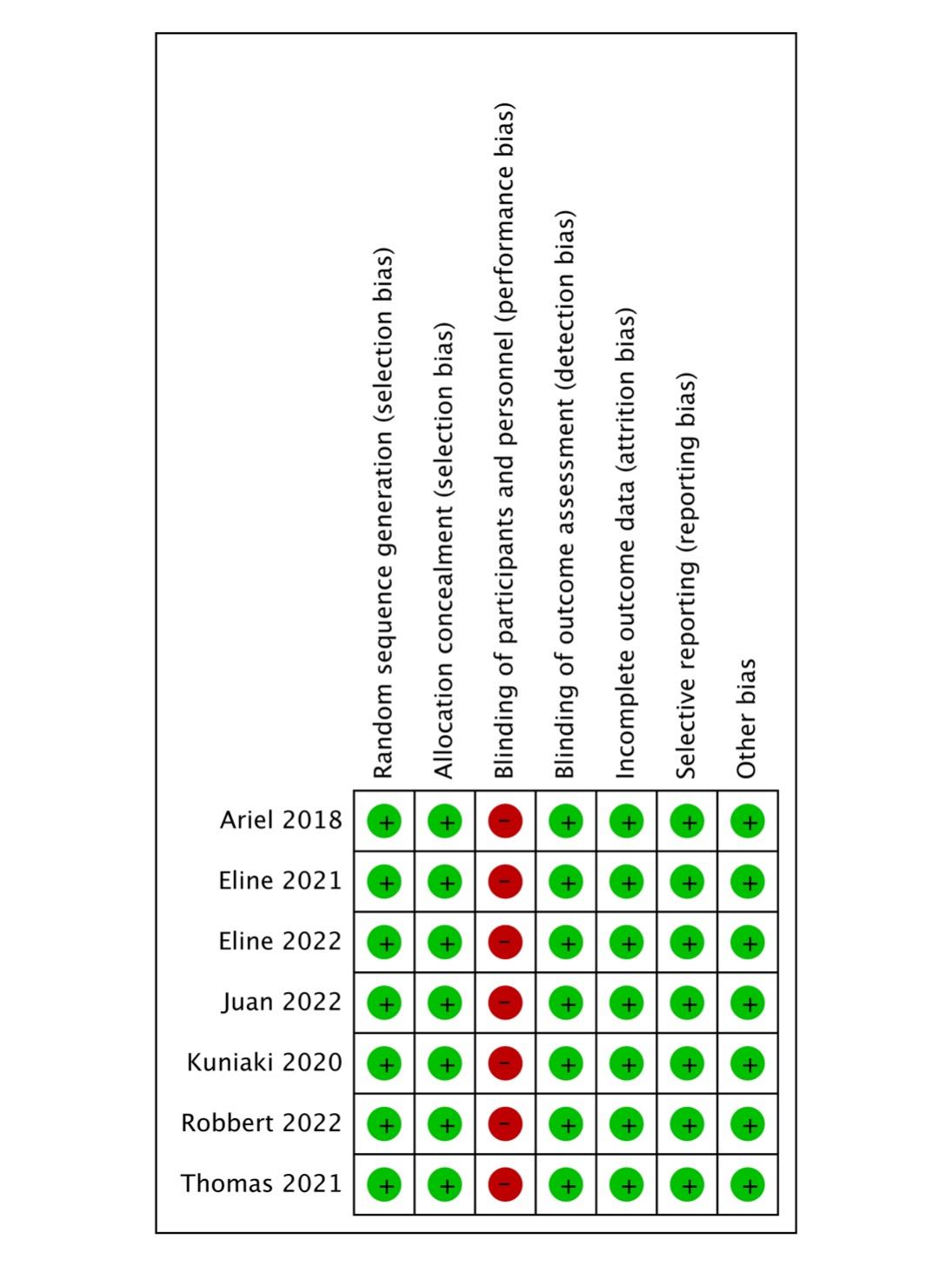


Figure S1. Risk of bias summary for included studies


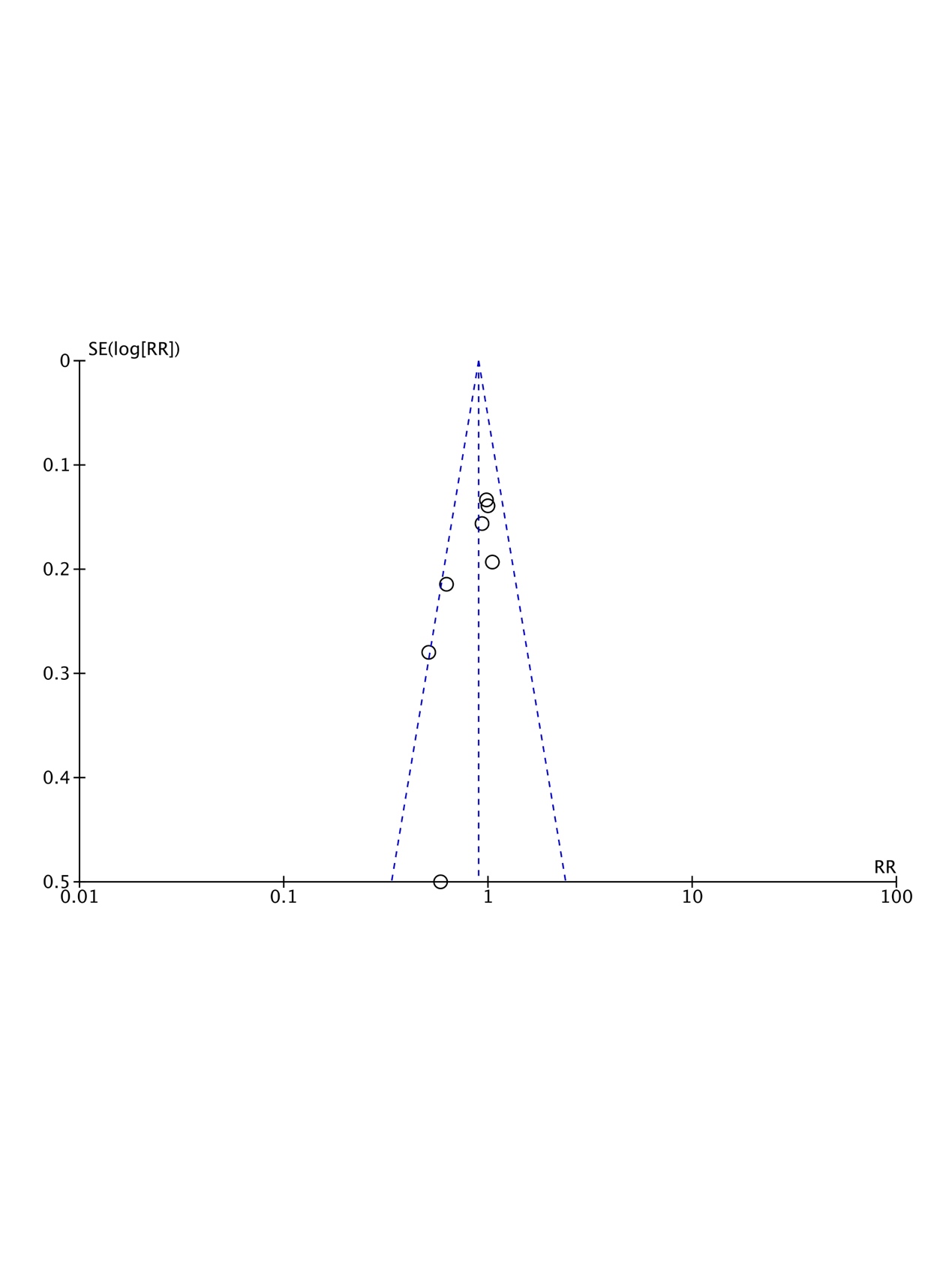


Figure S2. Funnel plot for publication bias for target lesion failure

1. Supplementary Tables

## Table S1 Entry and Exclusion criteria of each included study

| *Study* | *Entry criteria* | *Exclusion criteria* |
| --- | --- | --- |
| Robbert J. de Winter et al | 1. Patients aged at least 18 years. 2. Presence of one or more coronary artery stenoses of 50% or greater in a native coronary artery, saphenous venous graft, or arterial bypass conduit. 3. Reference vessel diameter ranging from 2.25 mm to 4.50 mm. | 1. Pregnant or nursing women. 2. Known contraindication or hypersensitivity to drug-eluting stents. 3. Recent PCI treatment within 6 months. 4. Life expectancy of less than 12 months due to concurrent medical conditions. 5. Inability or unwillingness to attend 1-month and 12-month follow-up clinics. 6. Currently enrolled in an ongoing trial yet to reach its primary endpoint. |
| Juan F. Iglesias et al | 1. CCS or ACS patients. 2. At least one >50% diameter de novo stenotic or in-stent restenotic lesion. 3. The lesion is located in a native coronary artery or a bypass graft suitable for stent implantation. | 1. Pregnant women. 2. Patients incapable of providing consent. 3. Patients currently participating in another trial before reaching its primary endpoint. 4. Patients intolerant to aspirin, clopidogrel, or components of drug-eluting stents. 5. Patients scheduled for surgery within 6 months of the index percutaneous coronary intervention, requiring discontinuation of dual antiplatelet therapy. |
| Eline H. Ploumen et al (2022) | 1. All-comer patients aged 18 years or older. 2. Patients with all coronary syndromes, de-novo and restenotic lesions, and coronary artery or bypass lesions. | 1. Participation in another randomized drug or device study before reaching its primary endpoint. 2. Planned surgery that would necessitate interrupting dual antiplatelet therapy within the first 6 months. 3. Known intolerance to components of the investigational product or required medication. 4. Uncertainty about the ability to adhere to follow-up procedures or an assumed life expectancy of less than a year. 5. Known pregnancy. |

## Table S1 Cont.

| *Study* | *Entry criteria* | *Exclusion criteria* |
| --- | --- | --- |
| Eline H. Ploumen et al (2021) | Patients requiring percutaneous coronary intervention for any coronary syndrome, with lesions of any type, length, or size, and involving any number of lesions or vessels. | 1. Participation in another randomized trial involving cardiovascular devices or antithrombotic/anticoagulant therapy before reaching the primary endpoint. 2. Known intolerance to components of the study drug-eluting stent (DES), or antithrombotic and/or anticoagulant therapy leading to non-adherence to any dual antiplatelet therapy (DAPT). 3. Planned elective surgical procedure requiring discontinuation of DAPT within the first 3 months after randomization. 4. Known pregnancy. 5. Unlikely adherence to scheduled follow-up or assumed life expectancy less than 1 year. |
| Thomas Pilgrim et al | 1. Patients with ST-segment elevation myocardial infarction (STEMI). 2. Referred for percutaneous coronary intervention (PCI) within 24 hours of symptom onset. 3. Presence of at least 1 culprit coronary lesion suitable for stent implantation. | 1. Patients with acute myocardial infarction (MI) caused by stent thrombosis. 2. Patients with mechanical complications. |
| Kuniaki Takahashi et al | 1. Patients aged at least 18 years who underwent percutaneous coronary intervention. 2. Lesion with a reference vessel diameter of 2.50-3.75 mm. | 1. Individuals who are pregnant or breastfeeding at the time of randomization. 2. Known contraindication or hypersensitivity to components of drug-eluting stents. 3. Presence of a concurrent medical condition with a life expectancy of less than 12 months. 4. Unwillingness or inability to attend outpatient clinics for 1-month and 12-month follow-up. 5. Participation in another ongoing trial that has not yet reached its primary endpoint. |
| Ariel Roguin et al | 1. Patients aged at least 18 years diagnosed with ischemic heart disease. 2. Patients undergoing planned stent implantation in de novo native coronary lesions. 3. Maximum of 3 coronary artery lesions. 4. Maximum of 2 native target vessels. | 1. Patients with STEMI within the past 72 hours. 2. Left ventricular ejection fraction (LVEF) < 30%. 3. Active stent thrombosis. 4. Creatinine clearance < 30 mL / min. 5. Any previous PCI within 9 months or within 30 days involving the target vessel. 6. Unlikely adherence to dual antiplatelet therapy. |

**Table S2.** Stent characteristics

| ***Stent Name*** | ***Durable / Bioabsorbable Polymer*** | ***Manufacturer*** | ***Strut Thickness*** | ***Metallic Platform*** | ***Polymer Thickness*** | ***Anti-proliferative Drug*** |
| --- | --- | --- | --- | --- | --- | --- |
| Orsiro | Bioabsorbable | Biotronik | 60 µm | Cobalt-chromium | Abluminal: 7.5 µm  Luminal: 3.5 µm | Sirolimus |
| MiStent | Bioabsorbable | MiCell Technologies | 64 μm | Cobalt-chromium | Abluminal: 15 μm  Luminal: 5 μm | Sirolimus |
| Supraflex | Bioabsorbable | APC Cardiovascular | 60 μm | Cobalt-chromium | 4 - 5 μm | Sirolimus |
| Xience Prime/Xpedition | Durable | Abbott | 81 µm | Cobalt-chromium | 7.6 µm | Everolimus |
| Resolute Integrity | Durable | Medtronic | 91 µm | Cobalt-chromium | 6 µm | Zotarolimus |
| Resolute Onyx | Durable | Medtronic | 81 µm | Cobalt-chromium | 5.6 µm | Zotarolimus |

**Table S3.** Interactions between stent type and clinical presentation for the risk of target lesion failure in origin studies

| ***Study*** | ***P for interaction**** |
| --- | --- |
| Robbert J. de Winter et al | 0.44 |
| Juan F. Iglesias et al | 0.77 |
| Eline H. Ploumen et al 2022 | 0.41 |
| Eline H. Ploumen et al 2021 | 0.74 |
| Thomas Pilgrim et al | - |
| Kuniaki Takahashi et al | 0.24 |
| Ariel Roguin et al | 0.24 |

*P-value for interaction that extracted from origin studies, describing interaction between stent type (ultrathin bioabsorbable-polymer sirolimus-eluting stents or thin durable-polymer drug-eluting stents) and clinical presentation (acute coronary syndrome or not)

**Table S4.** Risk of bias assessment

| ***Study (Author)*** | ***Random sequence generation*** | ***Allocation concealment*** | ***Blinding of participants & personnel*** | ***Blinding of outcome assessment*** | ***Incomplete outcome data*** | ***Selective reporting*** | ***Overall Quality*** |
| --- | --- | --- | --- | --- | --- | --- | --- |
| Robbert J. de Winter et al | Low risk | Low risk | High risk | Low risk | Low risk | Low risk | High |
| Juan F. Iglesias et al | Low risk | Low risk | High risk | Low risk | Low risk | Low risk | High |
| Eline H. Ploumen et al 2022 | Low risk | Low risk | High risk | Low risk | Low risk | Low risk | High |
| Eline H. Ploumen et al 2021 | Low risk | Low risk | High risk | Low risk | Low risk | Low risk | High |
| Thomas Pilgrim et al | Low risk | Low risk | High risk | Low risk | Low risk | Low risk | High |
| Kuniaki Takahashi et al | Low risk | Low risk | High risk | Low risk | Low risk | Low risk | High |
| Ariel Roguin et al | Low risk | Low risk | High risk | Low risk | Low risk | Low risk | High |

Table S5. Results of sensitivity analysis for the risk of target lesion failure by removing each trial in turn

| ***Trial removed*** | ***Relative Risk (95% CI)*** | ***P value*** |
| --- | --- | --- |
| Robbert J. de Winter et al | 0.88 (0.73, 1.06) | 0.17 |
| Juan F. Iglesias et al | 0.83 (0.67, 1.03) | 0.09 |
| Eline H. Ploumen et al 2022 | 0.83 (0.67, 1.04) | 0.10 |
| Eline H. Ploumen et al 2021 | 0.84 (0.67, 1.05) | 0.13 |
| Thomas Pilgrim et al | 0.92 (0.78, 1.09) | 0.34 |
| Kuniaki Takahashi et al | 0.84 (0.68, 1.02) | 0.08 |
| Ariel Roguin et al | 0.93 (0.81, 1.07) | 0.31 |
